# Six-Year Outcomes Following Stereotactic Body Radiation Therapy for Insulinoma: A Case Report and Literature Review

**DOI:** 10.7759/cureus.83535

**Published:** 2025-05-05

**Authors:** Abdullah Alswilem, Khaled K Hassan, Firas Almomen, Batoul Alruhaimi, Amna Mohaimeed, Noura Almazrou, Saif Aljabab, Yasir Alayed, Eyad Alsaeed, Abdullah Alsuhaibani

**Affiliations:** 1 Oncology Center, King Saud University Medical City, Riyadh, SAU; 2 Kasr Al-Ainy Center of Clinical Oncology, Faculty of Medicine, Cairo University, Cairo, EGY; 3 Radiation Oncology Unit, College of Medicine, King Saud University, Riyadh, SAU

**Keywords:** curative treatment, insulinoma, non-surgical management, pancreatic neuroendocrine tumor (pnet), stereotactic body radiation therapy (sbrt)

## Abstract

Insulinomas are the most prevalent hormone-producing pancreatic neuroendocrine neoplasms (panNEN). Surgical resection of these tumors is frequently curative and is currently the mainstay treatment for preventing long-term brain damage caused by hypoglycemia. Stereotactic body radiation therapy (SBRT) is an external radiation therapy utilizing specific equipment for positioning patients and delivering a focused radiation dose to tumors in the body over a series of sessions while fostering minimal radiation exposure to surrounding healthy tissue. This report presents a six-year follow-up and comprehensive update of a previously published case of insulinoma treated with SBRT, including new imaging, biochemical outcomes, detailed SBRT planning methodology, and an updated literature review. These additions provide long-term insight into SBRT as a high-precision alternative for patients who decline or are unfit for surgery.

## Introduction

Insulinomas are the most prevalent, hormone-producing pancreatic neuroendocrine neoplasms (panNEN), with an annual incidence rate of 0.7-4 cases per million people [1]. Insulinomas are ordinarily more prevalent in female patients than male, with a peak incidence occurring in men's fifth decade of life and women's sixth decade [2]. Localizing these tumors is often challenging because their small size usually necessitates multiple imaging modalities. Therefore, the diagnosis of insulinomas is based on a combination of clinical features, symptoms, laboratory tests, and imaging, all of which help determine the tumor location and size [3]. Although medical treatment can be employed first, it is not a curative option. Surgical resection remains the gold-standard curative option for preventing neurological impairments caused by chronic hypoglycemia [4]. Stereotactic radiosurgery or image-guided robotics (CyberKnife) is a non-invasive method that uses robotic systems to deliver high ionizing radiation to a distinct target [5]. Currently, there is limited literature focused on the use of radiation treatment as an ablative treatment to control insulinoma. Rather, most of the available literature focuses on the treatment of carcinoid tumors and neuroendocrine tumors, not insulinomas. Moreover, these treatment options may not be appropriate for all patients, such as those who are at high risk for surgical procedures or even those who just outrightly decline surgery. For such patients, stereotactic body radiation therapy (SBRT) has emerged as a viable option within the last two decades, and it has increasingly become popular in more recent years.

We report a rare case of long-term control of insulinoma using SBRT in a patient who declined surgery. This manuscript expands on a previously published case report by Alsuhaibani et al. [6], which described the initial presentation and decision for SBRT but did not include treatment planning details or post-treatment outcomes. In addition, we present a six-year follow-up with newly documented clinical, radiological, and biochemical outcomes, as well as a detailed overview of the SBRT planning and delivery process. This includes immobilization setup, simulation protocol, motion adaptation, dose constraints, and planning parameters. An updated literature review has been incorporated to better reflect the current understanding of managing functional pancreatic neuroendocrine tumors (PNETs). Furthermore, this report corrects previously misreported demographic information, confirming that SBRT was initiated in January 2019 when the patient was 58 years old, based on institutional records.

## Case presentation

A 58-year-old male with hypertension, well controlled on medications, was also treated at another institution for Hodgkin lymphoma 35 years ago, involving the neck and mediastinum with chemotherapy and mantle field radiotherapy. Specific radiation dose and fractionation details were not available in the medical records. The patient reported that for four years, he had had several hypoglycemic attacks discovered incidentally during admission for a transient ischemic attack (TIA). The multiple episodes of hypoglycemia prompted further evaluation.

A computed tomography (CT) scan of the abdomen at presentation was conducted, and imaging revealed a bulky 3.5 cm lesion in the uncinate process of the pancreas.

**Figure 1:**
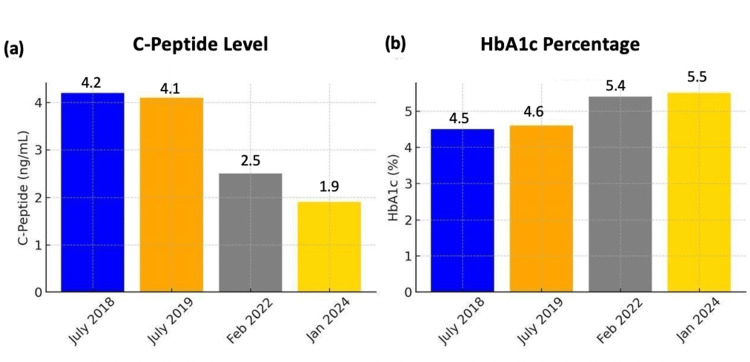
Biochemical investigation over time (C-peptide and HbA1c) (a) After SBRT, C-peptide levels steadily declined from 4.2 ng/mL in 2018 to 1.9 ng/mL by 2024, falling within the normal range (0.5-2.0 ng/mL). (b) HbA1c values increased slightly over time, rising from 4.5% in 2018 to 5.5% in 2024, but remained within the non-diabetic range (normal <5.7%). SBRT: Stereotactic body radiation therapy; HbA1c: Glycated hemoglobin

**Figure 2:**
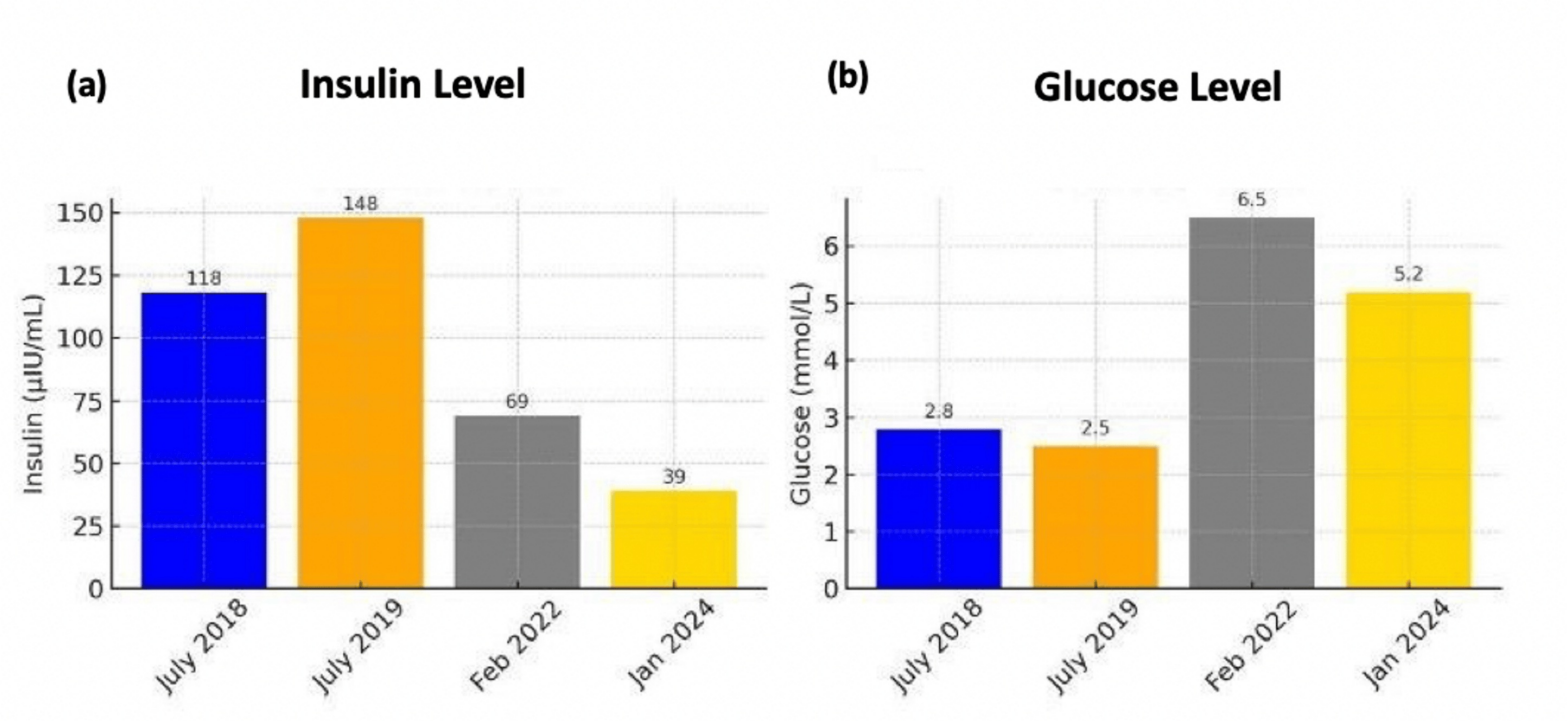
Biochemical investigation over time (insulin and glucose) (a) Insulin levels peaked in 2019 at 148 µIU/mL before steadily declining to 39 µIU/mL by 2024, closer to the normal range (2-20 µIU/mL) following SBRT. (b) Fasting glucose levels showed marked improvement, rising from hypoglycemic values in 2019 (2.5 mmol/L) to 5.2 mmol/L in 2024, within the normal range (3.9-5.6 mmol/L). SBRT: Stereotactic body radiation therapy

Further, biochemical testing established that insulin levels were four times higher than the normal value, serum blood glucose levels were low, and C-peptide levels were unusually high (Figures 1-2). Subsequently, an insulinoma diagnosis was established based on the biochemical and radiological findings in July 2018.

Following the diagnosis, the case was evaluated by an endocrinologist, a hepatobiliary surgeon, and a radiation oncologist. It was then discussed in a multidisciplinary tumor board meeting, and a consensus was reached to prepare the patient for Whipple’s operation, a surgical procedure that comes with a high risk for morbidity and mortality. The patient refused surgery due to perceived risk and decided to go for radiation therapy.

In January 2019, the patient received definitive SBRT to a total dose of 40 Gy in four fractions on alternate days. This regimen was chosen to achieve optimal local control while minimizing exposure to adjacent gastrointestinal structures, based on institutional experience and published data. The patient was simulated and treated in the supine position, with arms raised above the head and supported on a wing board. Immobilization was achieved using an SBRT abdominal compression belt (QFix, Avondale, USA) to minimize respiratory motion. Multiple non-contrast CT planning scans were obtained at 2 mm slice thickness across different respiratory phases. A free-breathing CT was used for gross tumor volume (GTV) and organs at risk (OAR) delineation, while inhale and exhale scans were co-registered to create a motion-adapted GTV (iGTV), due to the unavailability of four-dimensional CT (4DCT) or breath-hold systems at our center. The planning target volume (PTV) was generated by adding 5 mm radial and 7 mm longitudinal margins to the iGTV to account for setup uncertainties. The treatment plan was created using the Eclipse treatment planning system (Varian Medical Systems, Palo Alto, USA) with the volumetric modulated arc therapy (VMAT) technique and 10 MV photons, prescribing 40 Gy in four fractions to the 80% isodose line, covering at least 95% of the PTV. Normal tissue constraints were respected, with priority given to the duodenum (V15 < 9 cc, V25 < 3 cc, V33 < 1 cc). Treatment was delivered on a TrueBeam linear accelerator (Varian Medical Systems, Palo Alto, USA) with daily image-guided radiotherapy (IGRT) using cone-beam CT. The patient completed SBRT without major complications. He was maintained on prophylactic antiemetics and proton pump inhibitors (PPIs), and experienced grade 1 nausea, which was managed conservatively and resolved without additional intervention.

**Figure 3:**
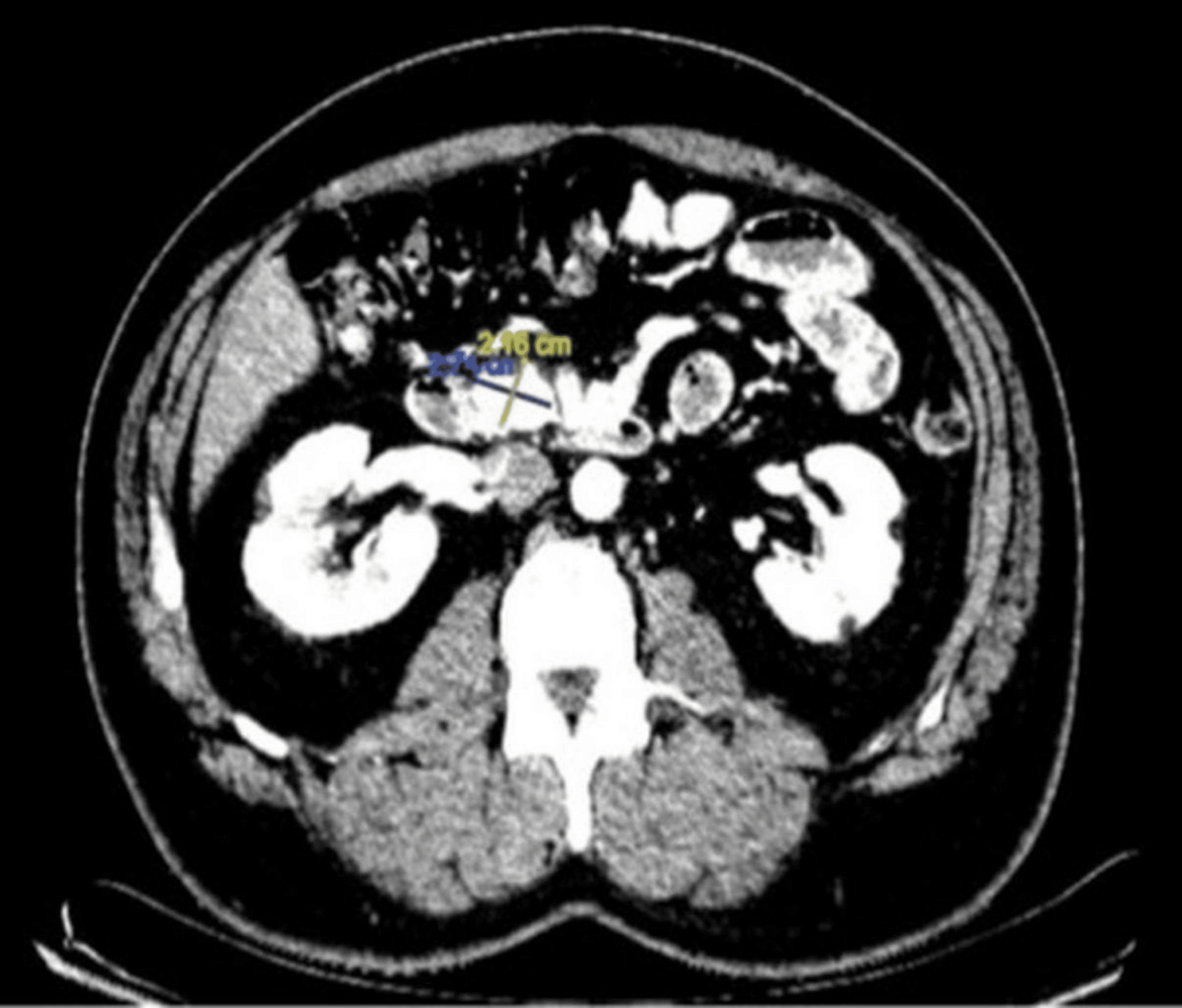
One-year follow-up CT scan CT scan obtained in December 2019, one year post-SBRT, showing tumor regression with reduced size of approximately 2.7 × 2.1 cm. CT: Computed tomography; SBRT: Stereotactic body radiation therapy

**Figure 4:**
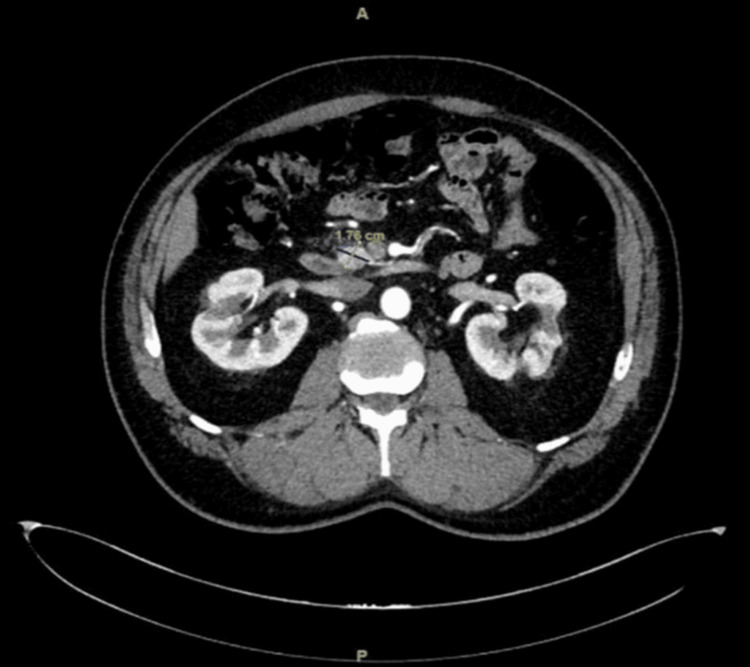
Three-year follow-up CT scan CT scan from February 2022, three years post-SBRT, demonstrating further reduction in tumor size to 2.2 × 1.8 cm. CT: Computed tomography; SBRT: Stereotactic body radiation therapy

**Figure 5:**
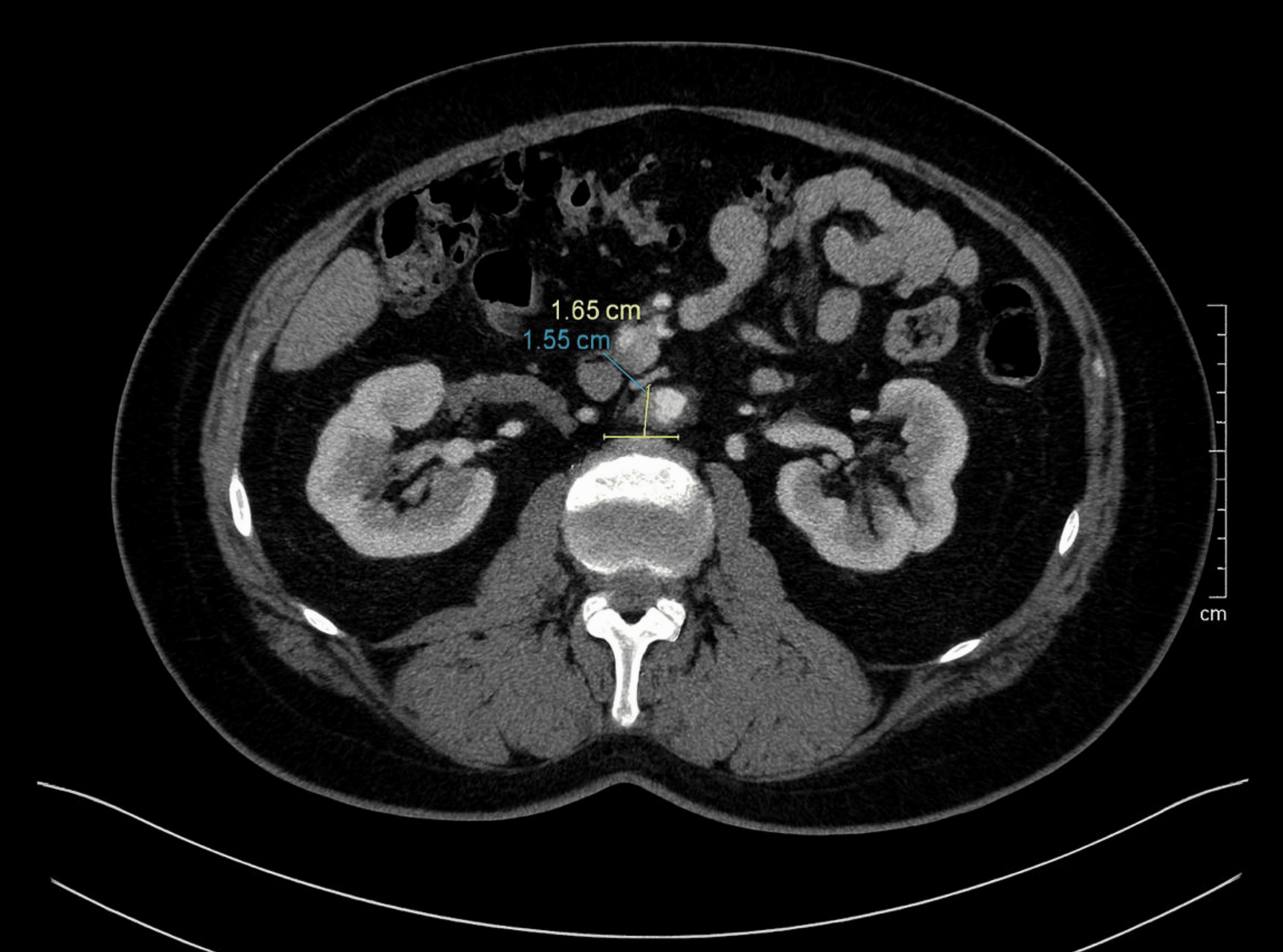
Six-year follow-up CT scan CT scan from January 2025, six years post-SBRT, showing continued tumor shrinkage with a residual lesion measuring 1.7 × 1.6 cm. CT: Computed tomography; SBRT: Stereotactic body radiation therapy

Since the treatment, the patient has been on regular follow-up, including clinical assessments, biochemical monitoring, and imaging. At six years post-SBRT, the patient was in sustained remission, and he had not experienced any episodes of hypoglycemia symptoms. He had not required glucose management interventions at any point during the six years post-SBRT. Moreover, no significant early or late radiation-induced toxicities were reported (Figures 3-5).

Serial fasting blood glucose monitoring confirmed that the patient maintained normoglycemia, with values consistently above 70 mg/dL (normal range: 70-100 mg/dL). Additionally, both insulin and C-peptide levels showed a progressive decline over time, reaching 39 µIU/mL (normal range: 2-20 µIU/mL) and 1.9 ng/mL (normal range: 0.5-2.0 ng/mL), respectively (Figures 1-2).

## Discussion

Hypoglycemia, or low blood sugar, became known as a clinical condition in the 19th century. On the other hand, clinical symptoms identical to those caused by insulin overdose were only discovered in non-diabetic patients in the early 1920s after insulin became accessible for the treatment of diabetes mellitus. The observation of symptoms attributable to excessive insulin led to the detection of a new medical condition known as hyperinsulinism [7]. In 1927, an autopsy performed on a patient with periods of severe hypoglycemia revealed the presence of a malignant pancreatic islet-cell tumor, thereby confirming the pathological basis of hyperinsulinism [8]. In 1929, the first effective cure for hyperinsulinism, which involved the surgical resection of insulinomas, was reported [9].

One of the leading causes of hypoglycemia is a rare PNET called insulinoma. This tumor produces insulin independently, with excess insulin leading to hyperinsulinemia and hypoglycemia [10]. Most insulinomas occur in a solitary form, and about 90% of them are usually non-malignant, with only about 10% being malignant [11]. Insulinomas primarily occur spontaneously, but it is important to note that about 5% to 10% of cases are associated with multiple endocrine neoplasia type 1 (MEN1), a relatively rare hereditary syndrome characterized by the development of neoplasms in a variety of endocrine organs, such as the pituitary gland, parathyroid glands, and pancreas [12]. Conversely, the prevalence of insulinoma among individuals with MEN1 is estimated to be about 10% [13]. Thus, a significant association exists between MEN1 and insulinoma, meaning that patients who are diagnosed with one of the conditions should consider screening for the other.

The clinical presentation of insulinomas is fairly variable and, therefore, can be difficult to diagnose because they do not have symptoms that exclusively identify them. Nevertheless, common symptoms of insulinomas include confusion, visual disturbances, behavioral abnormalities, dizziness, seizures, weakness, and loss of consciousness, all of which are secondary to hypoglycemia [10]. These symptoms are observed in patients for a period of 12-18 months, at which point a definite diagnosis can be reached [14]. Insulinomas are characterized by the presence of Whipple's triad, which includes hypoglycemia (a plasma glucose level of 50 mg/dL or lower). Neuroglycopenic symptoms, caused by reduced glucose availability to the brain, can manifest as confusion, blurred vision, difficulty concentrating, behavioral changes, memory loss, seizures, and, in severe cases, loss of consciousness. Relief of symptoms occurs after the administration of a glucose injection.

Henquin et al. characterized insulin release into three different categories based on the rate of insulin secretion: Group A, Group B, and Group C. The first category, Group A, describes insulinomas that exhibit a normal response to glucose, leucine, diazoxide, extracellular calcium chloride, and tolbutamide. Group B consists of insulinomas that react strongly to glucose, releasing large amounts of insulin and causing severe hypoglycemia. Lastly, insulinomas in the Group C category have considerably low insulin release rates despite hypoglycemia [15].

Whipple’s triad forms the primary basis upon which the process of diagnosing insulinoma begins. Therefore, the symptoms that characterize the clinical presentation of insulinoma must first be confirmed before clinical diagnosis. As for the clinical diagnosis itself, the gold standard is the 72-hour fasting protocol that tests for plasma concentrations of glucose, C-peptide, insulin, and proinsulin at the start of symptomatic hypoglycemia. In addition, for increased precision in locating the tumor, several imaging modalities can be utilized, such as CT, endoscopic ultrasonography (EUS), and magnetic resonance imaging (MRI). Accessibility to such modalities can vary between medical facilities, but they all have high sensitivity for insulinoma, with McAuley et al. reporting 94% sensitivity [16].

Surgical resection has been the main way of treating insulinoma for many decades, especially since the Whipple procedure was developed in the mid-20th century. Enucleation and partial distal pancreatectomy have been widely recognized as the two main types of surgery for treating insulinomas. Enucleation is ideal for small, localized insulinomas, and partial distal pancreatectomy is best for larger, diffuse insulinomas [17]. Laparoscopic or keyhole surgery is another surgical approach to treating insulinoma, and it is often preferred because it is minimally invasive [18]. In cases in which surgery cannot be conducted, medical therapy with diazoxide and/or analogs of somatostatin can represent alternative options for hypoglycemia management. In addition, newer modalities such as robotic enucleation and endoscopic ultrasound-guided ablation have proven effective in specific cases [19-21].

More recently, with SBRT, professionals administer high-dose per fraction therapy for a range of solid tumors with high accuracy and minimal damage to other tissues. Besides, SBRT is beneficial in that it can be delivered in 1-5 fractions, and it reduces interruptions in systemic therapy. Research has also shown that SBRT is generally well-tolerated, with patients experiencing minimal toxicities [22].

The current case involves a patient who underwent radiotherapy for the pancreatic tumor with a total dose of 40 Gy in four fractions, which was completed in January 2019 with good tolerance. As illustrated in the case report, the use of SBRT enabled the patient to achieve sustained remission with minimal symptoms of hypoglycemia, progressive decline in insulin and C-peptide levels to within normal limits, and sustained reduction in tumor size. Notably, there has been limited prior literature reporting the application of SBRT in treating insulinoma.

In an investigation by Ahmed et al. to assess radiosensitivity connected with response to liver metastases after SBRT, results indicated that small bowel neuroendocrine tumors had high radiosensitivity, whereas pancreatic and large bowel neuroendocrine tumors were predicted to have similar radiosensitivity to colorectal metastases [23]. Elsewhere, Bignardi et al. evaluated the impact of SBRT on two patients with liver metastases who were treated at doses of 45-60 Gy. According to Bignardi et al., the treatment achieved local control that lasted for more than 40 months [24]. SBRT has emerged as a highly favorable treatment for insulinoma, particularly for patients ineligible for surgical intervention [25-27]. Huscher et al., who documented the first case report using SBRT for the treatment of insulinoma, reported excellent symptom management over a three-year period [25]. Additionally, Huscher et al. reported that robotic radiosurgery, facilitated through real-time imaging guidance, is also an effective modality for high-risk patients whose tumors are ineligible for surgery [25].

In another study that has reported on the effectiveness of SBRT in treating insulinomas, Myrehaug et al. evaluated two different cases. In the first case, the patient was treated with a dose of 45 Gy over five fractions due to perioperative risks. The patient attained a stable glucose level (6.4-8.4 mmol/L) within one month after treatment. Moreover, the patient did not experience any associated toxicities, and the patient maintained glycemic control for 18 months [26]. In the second case, Myrehaug et al. reported treating a patient diagnosed with MEN1 who had two pancreatic lesions, each of which was treated with a dose of 30 Gy in three fractions. In this case, the patient did not experience any associated toxicities and reached a normal blood sugar level two months post-therapy. In addition, there was a substantial shrinking of the tumor and sustained response to therapy 28 months post-radiotherapy [26].

In a more recent publication, Namysl-Kaletka et al. (2024) reported two additional cases of insulinoma treated with SBRT using differing dose regimens: 25 Gy in five fractions and 30 Gy in three fractions. Both patients achieved biochemical and symptomatic improvement without significant toxicity. Their findings further support the feasibility and effectiveness of SBRT in managing functional PNETs, highlighting its value in non-surgical candidates and contributing to a more robust evidence base for this modality [27].

The original report by Alsuhaibani et al. [6] focused on the initial diagnosis and treatment decision but did not include post-treatment outcomes or technical details of SBRT. In contrast, the current manuscript presents a comprehensive six-year follow-up, with sustained tumor control, biochemical remission, and a detailed SBRT methodology. This includes immobilization setup, simulation protocol, motion adaptation, dose constraints, and planning parameters. An updated literature review has also been incorporated to provide context for the evolving role of SBRT in managing functional PNETs. These additions deliver both scientific depth and educational value, offering novel insight into the long-term, non-surgical management of insulinoma.

This case adds to the growing clinical experience supporting SBRT as a safe and effective option in the management of insulinoma, particularly for patients who are not surgical candidates. The six-year outcome reinforces the durability of tumor control and long-term symptom relief with minimal toxicity. Moving forward, larger prospective studies and standardized treatment protocols are needed to further define the role of SBRT in functional PNETs and to ensure consistent, high-quality outcomes.

## Conclusions

Presently, surgical resection is still the main and universal modality of treating insulinomas. However, radiation therapy can become a necessity in cases of patients who, for one reason or another, cannot receive surgical intervention. SBRT is a non-invasive modality that is effective in controlling symptoms and tumors, giving it some advantages over traditional surgical resection. However, a multidisciplinary approach must be taken when deciding to use SBRT to treat a particular patient in practice. Doing so makes it feasible to apply personalized treatment plans that maximize patient outcomes.
